# Prognostic value of cachexia index in patients with colorectal cancer: A retrospective study

**DOI:** 10.3389/fonc.2022.984459

**Published:** 2022-09-23

**Authors:** Qianyi Wan, Qian Yuan, Rui Zhao, Xiaoding Shen, Yi Chen, Tao Li, Yinghan Song

**Affiliations:** ^1^ Department of Gastrointestinal Surgery, West China Hospital, Sichuan University, Chengdu, China; ^2^ Operating Room of Anesthesia Surgery Center, West China Hospital/West China School of Nursing, Sichuan University, Chengdu, China; ^3^ Laboratory of Mitochondria and Metabolism, Department of Anesthesiology, National Clinical Research Center for Geriatrics, West China Hospital, Sichuan University, Chengdu, China; ^4^ Department of day surgery center, West China Hospital, Sichuan University, Chengdu, China

**Keywords:** colorectal cancer, cachexia index, cancer cachexia, major complications, overall survival

## Abstract

**Background:**

Current diagnostic criteria for cancer cachexia are inconsistent, and arguments still exist about the impact of cachexia on the survival of patients with colorectal cancer. In this study, we aim to investigate the prognostic value of a novel cachexia indicator, the cachexia index (CXI), in patients with colorectal cancer.

**Methods:**

The CXI was calculated as skeletal muscle index (SMI) × serum albumin/neutrophil-lymphocyte ratio. The cut-off value of CXI was determined by the receiver operating characteristic (ROC) curves and Youden’s index. The major outcomes were major complications, overall survival (OS), and recurrence-free survival (RFS).

**Results:**

A total of 379 patients (234 men and 145 women) were included. The ROC curves indicated that CXI had a significantly diagnostic capacity for the detection of major complications. Based on Youden’s index, there were 231 and 148 patients in the low and high CXI groups, respectively. Patients in the low CXI group had significantly older age, lower BMI, and a higher percentage of cachexia and TNM stage II+III. Besides, Patients in low CXI group were associated with a significantly higher rate of major complications, blood transfusion, and longer length of stay. Logistic regression analysis indicated that low CXI, cachexia, and coronary heart disease were independent risk factors for the major complications. Kaplan Meier survival curves indicated that patients with high CXI had a significantly more favorable OS than those with low CXI, while no significant difference was found in RFS between the two groups. Besides, there were no significant differences in OS or RFS between patients with and without cachexia. The univariate and multivariate Cox regression analysis indicated that older age, low CXI, and coronary heart disease instead of cachexia were associated with a decreased OS.

**Conclusion:**

CXI was better than cachexia in predicting OS and could be a useful prognostic indicator in patients with colorectal cancer, and greater attention should be paid to patients with low CXI.

## Introduction

Cancer cachexia is a multifactorial syndrome, and its key characteristic is the loss of skeletal muscle mass (1). It is driven by various metabolic changes such as increased energy expenditure, excess catabolism, and elevated inflammation (2). Colorectal cancer is the third most commonly diagnosed cancer worldwide, and it ranks second in the cause of cancer death because of its high fatality rate (3). Cachexia is highly prevalent in colorectal cancer. Based on the diagnostic criteria that weight loss over 5% in the previous 6 months, the prevalence of cachexia in colorectal cancer is nearly 50% (2).

Current diagnostic criteria for cancer cachexia are inconsistent across studies (4). One of the most accepted international consensus in 2011, the Fearon criteria, recommended a combination of weight loss, body mass index (BMI), and skeletal muscle index (SMI) for the diagnosis of cancer cachexia, in which an accurate estimate of weight loss is indispensable (1). However, weight loss is not a completely objective indicator because not all patients can provide an accurate estimate of weight loss over the past 6 months, which could increase the risk of recalling bias. There are few studies investigating the impact of cancer cachexia on the survival of patients with colorectal cancer, and the results are inconsistent. For example, Gannavarapu et al. and Thoresen et al. indicate that cachexia is a poor prognostic factor for patients with colorectal cancer (5, 6). While Shibata et al. suggest that cachexia couldn`t completely predict the survival in patients with colorectal cancer (7). Furthermore, a cohort study by Brown et al. finds that a stable body weight does not mean that there is no loss of skeletal muscle in colorectal cancer (8). As a consequence, arguments still exist for the diagnostic criteria of cachexia between clinical assessment and the Fearon criteria in patients with colorectal cancer (9, 10).

Cachexia index (CXI), a new measure of cachexia, is consisted of three objective indicators including SMI, serum albumin, and neutrophil-lymphocyte ratio (NLR) (11). Recent studies have indicated that CXI is significantly associated with treatment response and prognosis in patients with malignancies such as lung cancer, liver cancer, and aggressive lymphomas (11–15). In this study, we aim to evaluate the prognostic value of CXI in patients with colorectal cancer, which might be helpful to distinguish the potentially cachexic patients.

## Methods

### Patients

Patients with colorectal cancer undergoing radical surgery at the Department of Gastrointestinal Surgery of West China Hospital from October 2020 to September 2021 were retrospectively collected in this study. The inclusion criteria for the patients were (1): pathology confirmed colorectal cancer (2); between 18 and 80 years old; (3) the preoperative CT scan was performed in our hospital. Besides, the exclusion criteria were: (1) patients undergoing emergency or non-radical surgery; (2) having a history of other malignancies. All the clinicopathological data were collected from the medical records, examination reports, and pathological reports through the Hospital Information System (HIS) of West China Hospital. All patients were routinely followed up after surgery, and the last follow-up data were collected in May 2022. All the included patients were anonymized before analysis. This study was conducted according to the Declaration of Helsinki and approved by the ethics committee of West China Hospital.

### Assessment of preoperative CXI, cachexia, and postoperative major complications

The preoperative CXI was calculated as the following formula: SMI (cm^2^/m^2^) × serum albumin (g/L)/NLR (11). The SMI was measured based on the preoperative abdominal CT images of included patients. The skeletal muscle area of the third lumbar vertebra (L3) level was analyzed using the software of syngo MultiModality Workplace (Siemens Medical Solutions, Forchheim, Germany) and BMI_CT (Seoul, South Korea), and the Hounsfield unit (HU) threshold for the skeletal muscle was set as -29 to 150 (16). The SMI was reported as total skeletal muscle area (cm^2^) of L3/height squared (m^2^) (17, 18). NLR was reported as the number of peripheral neutrophils/the number of peripheral lymphocytes (19). The postoperative major complications were recognized as surgical complications ≥ Grade III (20). Cachexia was diagnosed according to the Fearon criteria: weight loss > 5% over the past 6 months; or BMI < 20 and any degree of weight loss > 2%; or sarcopenia and any degree of weight loss > 2% (1). The L3 SMI cut-offs for sarcopenia were defined as <52.4 (men) and <38.5 cm^2^/m^2^ (women), respectively (21–24).

### Statistical analysis

A two-sided *P*-value of < 0.05 meant statistical significance in this study. For continuous data, the t-test or Mann–Whitney U test was used for comparison according to the normality, and the Chi-squared test or Fisher’s exact test was used for categorical data. To investigate the diagnostic capacity of CXI in the detection of major complications, receiver operating characteristic (ROC) curves were conducted. The cut-off value of CXI for defining the low and high CXI was determined according to Youden’s index. The univariate logistic regression analysis was applied for investigating the associations between multiple clinicopathological variables and the risk of major complications, and variables with a *P*-value of < 0.2 in the univariate analysis were further analyzed in multivariate analysis. Kaplan–Meier survival curves were used for analyzing the survival data, and the differences in survival curves were analyzed by log-rank tests. The univariate Cox proportional hazards model was also used for analyzing the overall survival (OS). Then, variables with a *P*-value of < 0.2 in the univariate analysis were further analyzed in multivariate analysis. The SPSS version 25.0 and GraphPad Prism version 8.0 were used for statistical analyses in this study.

## Results

A total of 379 patients (234 men and 145 women) with a mean age of 60.42 (± 11.06) years old were included in this study, and three patients were lost to follow-up. There were 136 patients with colon cancer and 243 with rectal cancer, respectively. 113 patients (29.82%) were diagnosed with cachexia based on the Fearon criteria. Postoperative pathology indicated that there were 94, 142, and 143 patients classified as Tumor-node-metastasis (TNM) stage I, II, and III, respectively. Besides, 24 patients (6.33%) had major surgical complications and 21 patients (5.54%) had a blood transfusion. Detailed clinical characteristics of included patients were shown in **Table 1**.

**Table 1:** Comparison of the clinical characteristics between patients in low and high CXI groups.

Characteristics	Total (n = 379)	Low CXI (n = 231)	High CXI (n = 148)	*P* value
Male/Female, n	234/145	131/100	103/45	0.01
Age, mean ± SD (years)	60.42 ± 11.06	61.36 ± 11.32	58.96 ± 10.52	0.04
CXI, mean ± SD	1052.52 ± 502.11	740.73 ± 224.25	1539.16 ± 422.38	< 0.001
BMI, mean ± SD	23.34 ± 3.12	22.97 ± 3.15	23.91 ± 2.97	0.004
Cachexia, n (yes/no)	113/266	80/151	33/115	0.01
Tumor site				0.001
Colon cancer	136	98	38	
Rectal cancer	243	133	110	
TNM stage, n				0.03
I	94	47	47	
II	142	95	47	
III	143	89	54	
Postoperative adjuvant chemotherapy, n (yes/no) ^*^	255/121	153/75	102/46	0.71
Cigarette smoking, n (yes/no)	58/321	29/202	29/119	0.06
Alcohol drinking, n (yes/no)	35/344	16/215	19/129	0.05
Hypertension, n (yes/no)	75/304	50/181	25/123	0.26
Coronary heart disease, n (yes/no)	15/364	8/223	7/141	0.54
Diabetes, n (yes/no)	38/341	23/208	15/133	0.96
Major complication, n (yes/no)	24/355	22/209	2/146	0.001
Blood transfusion, n (yes/no)	21/358	20/211	1/147	0.001
Length of stay, mean ± SD (days)	8.27 ± 3.81	8.68 ± 4.33	7.63 ± 2.70	0.01

BMI, body mass index; CXI, cachexia index; SD, standard deviation; TNM stage, Tumor-node-metastasis stage.

^*^Three patients were lost to follow-up.

**Figure 1:**
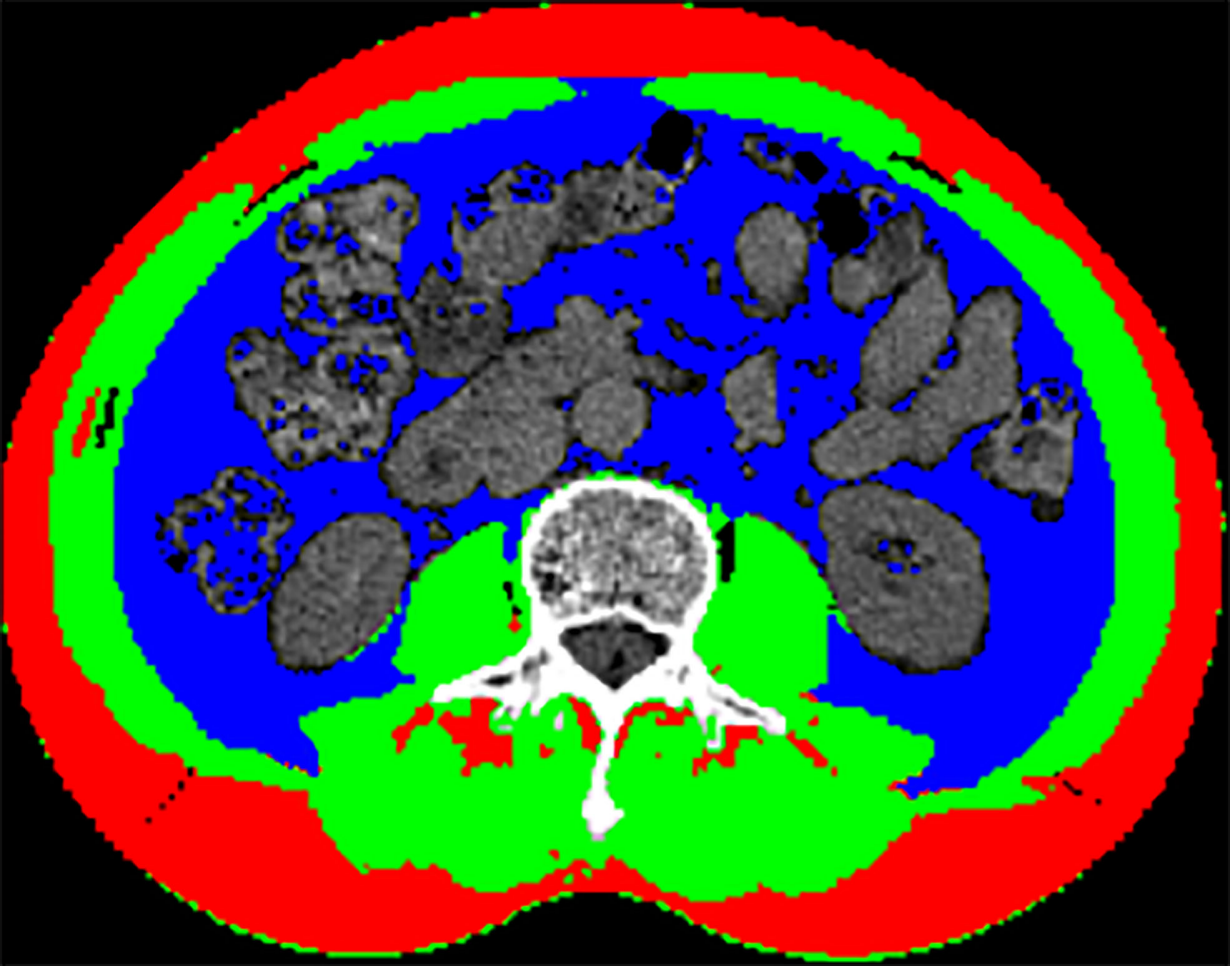
Representative CT image of the skeletal muscle area at L3 level: (Green) skeletal muscle; (Blue) visceral adipose tissue; (Red) subcutaneous adipose tissue.

**Figure 2:**
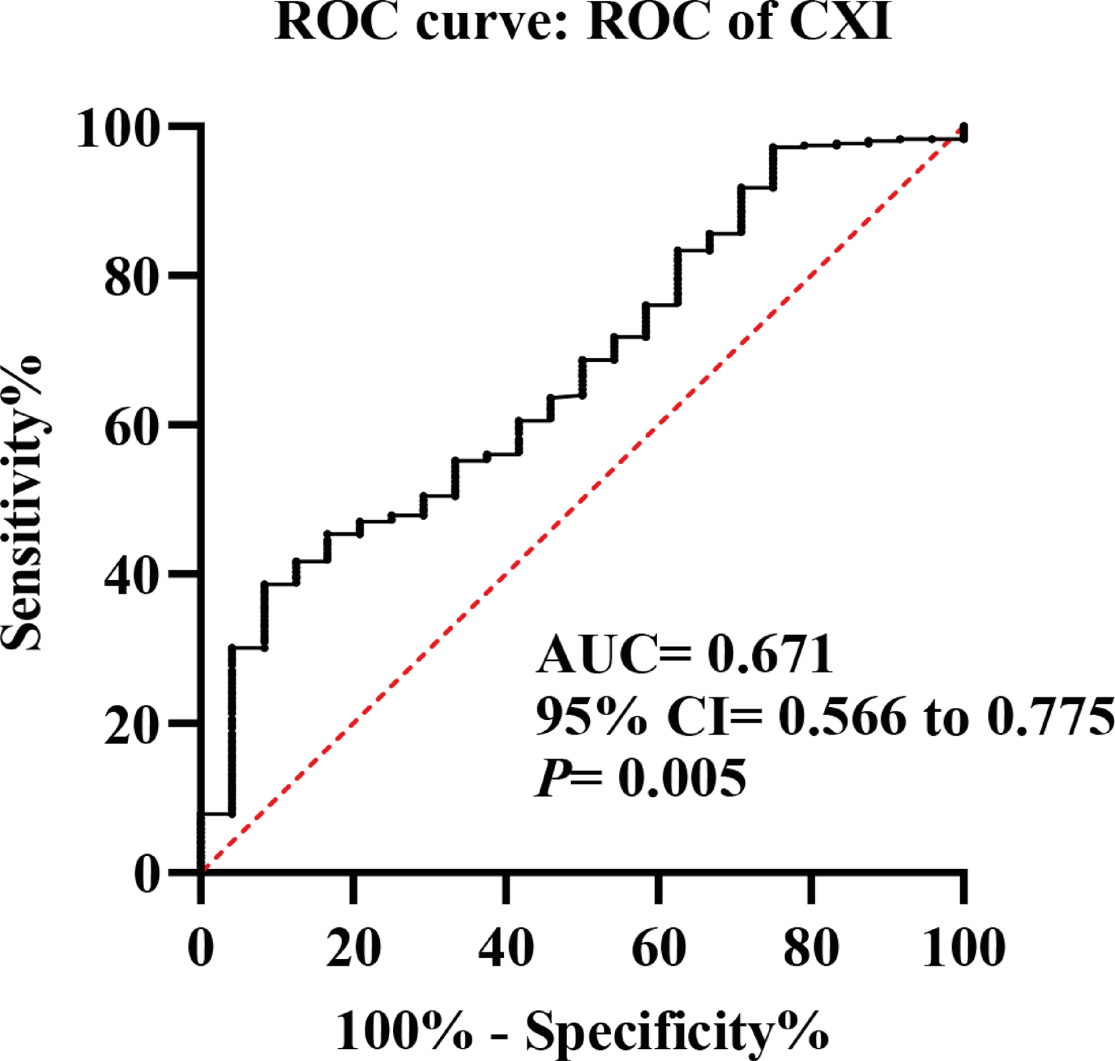
Receiver operating characteristic (ROC) curves of cachexia index (CXI) for the detection of major complications.

The representative CT image for assessing the skeletal muscle area of the L3 level was shown in **Figure 1**, and the mean CXI for all included patients was 1052.52 (± 502.11). Through ROC curve, we observed that CXI had a significantly diagnostic capacity for the detection of major complications (AUC: 0.671; 95%CI: 0.566 to 0.775; *P*= 0.005) (**Figure 2**).

Based on the ROC curves of major complications and Youden’s index, patients with a CXI of < 1087 (male) or < 1164 (female) were classified as low CXI group, and patients with a CXI of ≥ 1087 (male) or ≥ 1164 (female) were classified as high CXI group (**Figure S1**). Eventually, there were 231 patients with low CXI and 148 patients with high CXI, respectively. Patients in the low CXI group had a significantly older age (61.36 ± 11.32 vs. 58.96 ± 10.52, *P*= 0.04), lower BMI (22.97 ± 3.15 vs. 23.91 ± 2.97, *P*= 0.004), and a higher percentage of cachexia (*P*= 0.01) and TNM stage II+III (*P*= 0.03). Besides, Patients in low CXI group were associated with a significantly higher rate of major complications (9.52% vs. 1.35%, *P*= 0.001), blood transfusion (8.66% vs. 0.68%, *P*= 0.001), and longer length of stay (8.68 ± 4.33 vs. 7.63 ± 2.70, *P*= 0.01) (**Table 1**). No significant differences were found in postoperative adjuvant chemotherapy, cigarette smoking, alcohol drinking, hypertension, coronary heart disease, and diabetes between the two groups (**Table 1**).

In logistic regression analysis for the associations between multiple clinical variables and risk of major complications, both univariate and multivariate analysis indicated that low CXI (multivariate analysis: HR 0.14, 95% CI 0.03 to 0.63; *P*= 0.01), cachexia (multivariate analysis: HR 3.03, 95% CI 1.27 to 7.19; *P*= 0.01), and coronary heart disease (multivariate analysis: HR 4.73, 95% CI 1.09 to 20.57; *P*= 0.04) were independent risk factors for the major complications (**Table 2**).

**Table 2:** Univariate and multivariate analysis of major complications.

Characteristics	Univariate		Multivariate	
	HR (95% CI)	*P*	HR (95% CI)	*P*
Age > 60 years, (≤ 60 as ref)	1.74 (0.73 to 4.16)	0.22		
Female, (male as ref)	1.04 (0.44 to 2.43)	0.94		
BMI > 20, (≤ 20 as ref)	0.64 (0.23 to 1.78)	0.39		
CXI, (low CXI as ref)	0.13 (0.03 to 0.56)	0.01	0.14 (0.03 to 0.63)	0.01
Cachexia, (no as ref)	3.62 (1.56 to 8.42)	0.003	3.03 (1.27 to 7.19)	0.01
Tumor site, (colon cancer as ref)	0.93 (0.40 to 2.18)	0.87		
TNM stage III, (stage I and II as ref)	0.82 (0.34 to 1.96)	0.65		
Cigarette smoking, (no as ref)	0.78 (0.23 to 2.70)	0.69		
Alcohol drinking, (no as ref)	0.41 (0.05 to 3.14)	0.39		
Hypertension, (no as ref)	1.74 (0.69 to 4.36)	0.24		
Diabetes, (no as ref)	1.31 (0.37 to 4.60)	0.68		
Coronary heart disease, (no as ref)	4.08 (1.07 to 15.59)	0.04	4.73 (1.09 to 20.57)	0.04

BMI, body mass index; CXI, cachexia index; HR, hazard ratios; TNM stage, Tumor-node-metastasis stage; 95% CI, 95% confidence intervals.

**Figure 3:**
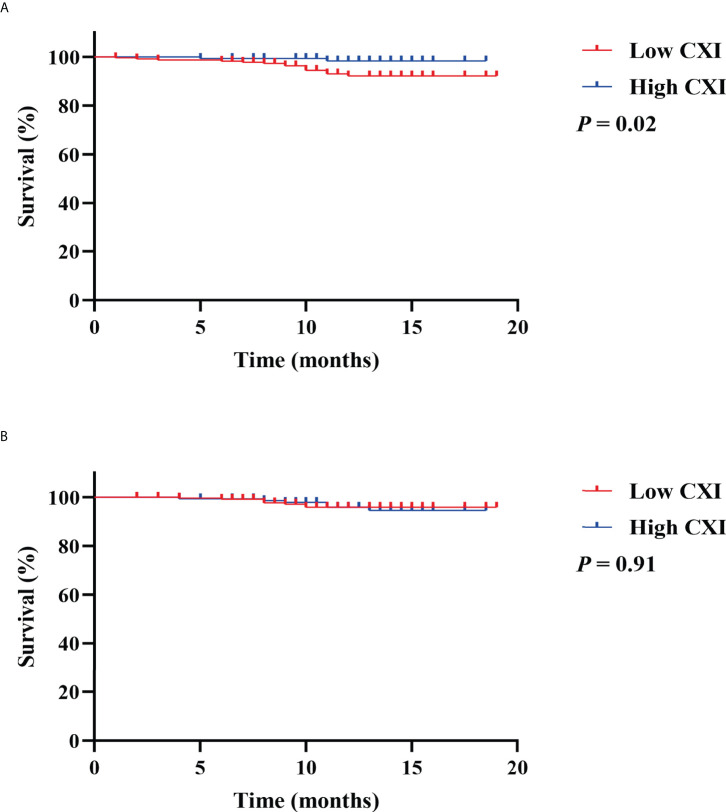
Kaplan Meier survival curves for the associations between cachexia index (CXI) and **(A)** overall survival; **(B)** recurrence free survival.

**Figure 4:**
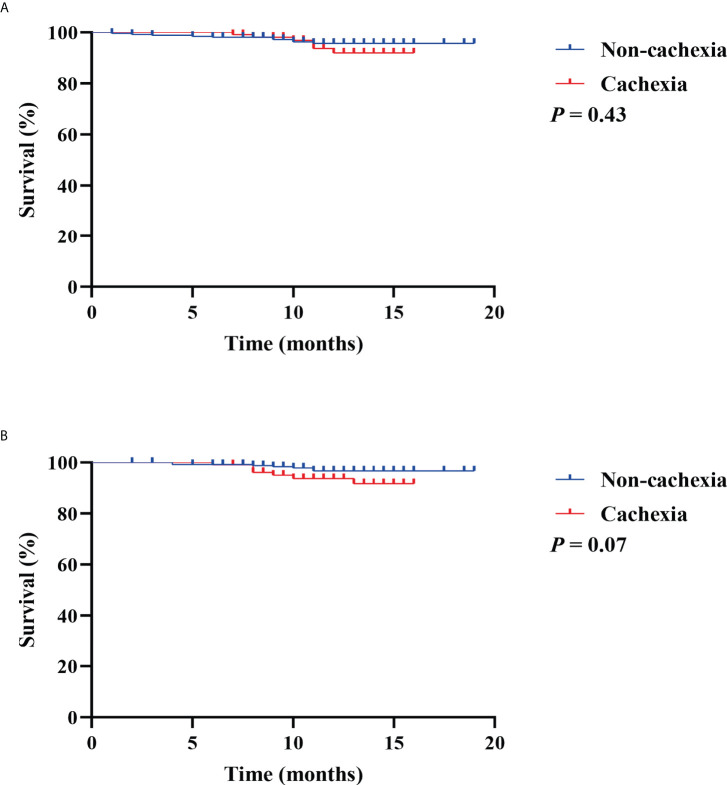
Kaplan Meier survival curves for the associations between cachexia and **(A)** overall survival; **(B)** recurrence free survival.

To investigate the prognostic value of CXI and cachexia in the survival of colorectal patients, the Kaplan–Meier survival analyses for OS and recurrence-free survival (RFS) were conducted. The results indicated that patients with high CXI had a significantly more favorable OS than those with low CXI (*P*= 0.02, **Figure 3A**), while no significant difference was found in RFS between the two groups (*P*= 0.91, **Figure 3B**). There were no significant differences in OS (*P*= 0.43, **Figure 4A**) or RFS (*P*= 0.07, **Figure 4B**) between patients with and without cachexia. Univariate and multivariate regression analysis indicated that older age (multivariate analysis: HR 5.22, 95% CI 1.17 to 23.16; *P*= 0.03), low CXI (multivariate analysis: HR 0.18, 95% CI 0.04 to 0.79; *P*= 0.02), and coronary heart disease (multivariate analysis: HR 5.78, 95% CI 1.60 to 20.86; *P*= 0.01) instead of cachexia were significantly associated with a decreased OS (**Table 3**).

**Table 3:** Univariate and multivariate analysis of overall survival.

Characteristics	Univariate		Multivariate	
	HR (95% CI)	*P*	HR (95% CI)	*P*
Age > 60 years, (≤ 60 as ref)	6.00 (1.36 to 26.38)	0.02	5.22 (1.17 to 23.16)	0.03
Female, (male as ref)	1.35 (0.47 to 3.89)	0.58		
BMI > 20, (≤ 20 as ref)	1.16 (0.26 to 5.11)	0.84		
CXI, (low CXI as ref)	0.20 (0.05 to 0.89)	0.03	0.18 (0.04 to 0.79)	0.02
Cachexia, (no as ref)	1.50 (0.54 to 4.12)	0.43		
Tumor site, (colon cancer as ref)	1.26 (0.44 to 3.64)	0.66		
TNM stage III, (stage I and II as ref)	1.52 (0.57 to 4.06)	0.40		
Postoperative adjuvant chemotherapy, (no as ref)	1.38 (0.45 to 4.28)	0.58		
Cigarette smoking, (no as ref)	0.37 (0.05 to 2.78)	0.33		
Alcohol drinking, (no as ref)	0.65 (0.09 to 4.91)	0.68		
Hypertension, (no as ref)	1.93 (0.67 to 5.54)	0.23		
Diabetes, (no as ref)	0.58 (0.08 to 4.36)	0.59		
Coronary heart disease, (no as ref)	5.96 (1.70 to 20.91)	0.01	5.78 (1.60 to 20.86)	0.01

BMI, body mass index; CXI, cachexia index; HR, hazard ratios; TNM stage, Tumor-node-metastasis stage; 95% CI, 95% confidence intervals.

## Discussion

In this study of 379 patients, we firstly investigated the prognostic value of CXI in patients with colorectal cancer. We found that a lower CXI was significantly associated with older age, lower BMI, and a more advanced TNM stage, which might reflect a more serious disease state. Besides, patients with low CXI were more likely to be diagnosed with cachexia, indicating that CXI could be a measurement of cachexia. Be similar to the previous studies that low CXI was an independent negative prognostic factor for OS in patients with lung cancer, liver cancer, and aggressive lymphomas (11–15), the Kaplan Meier survival curves and Cox regression analysis in this study also indicated that patients with high CXI had a significantly more favorable OS than those with low CXI. Besides, we found that CXI had no associations with the RFS, indicating that CXI is a prognostic indicator for OS instead of RFS. Furthermore, we first found that low CXI was significantly associated with poor perioperative outcomes, including a higher rate of major complications, blood transfusion, and longer length of stay. We also investigated the prognostic of cachexia diagnosed according to the Fearon criteria. However, Kaplan Meier survival curves and Cox regression analysis indicated that cachexia had no significant associations with the postoperative survival. These findings demonstrated that CXI was better than cachexia in predicting OS and could be a useful prognostic indicator in patients with colorectal cancer.

Cancer cachexia is widely recognized as a disorder characterized by an ongoing loss of skeletal muscle mass (regardless of the loss of fat mass) and cannot be fully reversed by conventional nutritional support (1, 2). Skeletal muscle loss, malnutrition, and increased inflammatory response are three key features of cancer cachexia (2, 4, 25). Current diagnostic criteria for cachexia mainly include the cancer-specific and general criteria (4). Although these diagnostic criteria of cancer cachexia have differences from each other, an estimate of weight loss is indispensable. However, weight loss as the main diagnostic criterion of cancer cachexia might increase the risk of recalling bias. Besides, a loss of total body weight cannot reflect a specific loss of skeletal muscle and fat. Furthermore, in some patients with advanced cancer, the fluid gains such as edema and malignant ascites could mask the actual weight loss (26, 27), which might increase the risk of bias when investigating cancer cachexia.

CXI is a new measure of cachexia that is calculated as SMI (cm^2^/m^2^) × serum albumin (g/L)/NLR, and these three parameters are objective and easily accessed from abdominal CT scans, routine peripheral blood, and biochemical tests. CT-determined SMI is a useful indicator to reflect the skeletal muscle mass and has been widely applied for investigating sarcopenia in many clinical studies (28–30). Our previous umbrella review also summarized that SMI-determined sarcopenia was significantly associated with multiple health-related outcomes in older populations and patients with or without tumors (31). An abdominal CT scan is a routine examination for patients with colorectal cancer before surgery, and CT-determined SMI of the L3 level is recognized as reflecting the whole-body muscle mass (32, 33). Therefore, CT-determined SMI is available for almost every patient with colorectal cancer and could be a key indicator for assessing the cancer cachexia. For malnutrition, albumin is an important nutritional marker in patients with gastrointestinal cancer (34, 35). It is reported that hypoalbuminemia can reflect cancer-induced malnutrition and have a negative impact on prognosis in patients with cancer (36, 37). NLR is an inflammation-related marker in the calculation of CXI, and it is also recognized as an indicator of cancer-related systemic inflammation in gastrointestinal cancer (36, 38–40). Therefore, the three objective indicators in the calculation formula of CXI could reflect the skeletal muscle status, malnutrition, and systematic inflammatory response of cancer cachexia, respectively.

Notably, the reality of CXI might be influenced by some drugs and diseases. In patients with diabetes, for example, the use of insulin could affect the level of serum albumin (41, 42). Besides, patients with liver cirrhosis usually have significantly decreased serum albumin (43). Furthermore, some studies suggest that the use of steroids could lead to an increase in NLR (44, 45). Therefore, the use of specific drugs should be noted when calculating CXI. Be limited by lacking relevant data, we did not investigate the impact of the use of specific drugs on the prognostic value of CXI in this study, and further studies about this issue are required in the future.

The strengths of this study are that we first investigate the prognostic value of CXI in patients with colorectal cancer, and our sample is the largest among the relevant studies about CXI and prognosis of malignancies. Our results indicate that low CXI is an independent negative prognostic factor for OS, and we also first suggest that low CXI was significantly associated with poor perioperative outcomes including a higher rate of major complications, blood transfusion, and longer length of stay. There are also several limitations of this study. Because this is a single-center and retrospective study, the risk of selection bias might be increased. Besides, it is the first study investigating CXI in colorectal cancer, thus, no external validation is available. The cut-off value for determining the low and high CXI needs further prospective studies to verify in the future. Furthermore, we only included Chinese patients in this study, and whether CXI could be a prognostic indicator for other races remains to be verified.

In conclusion, our study identified that CXI was better than cachexia in predicting OS and could be a useful prognostic indicator in patients with colorectal cancer, and greater attention should be paid to patients with low CXI. Considering the limitations of this study, our results need more prospective studies to verify in the future.

## Data availability statement

The original contributions presented in the study are included in the article/**Supplementary Material**. Further inquiries can be directed to the corresponding authors.

## Ethics statement

The studies involving human participants were reviewed and approved by the ethics committee of West China Hospital, Sichuan University. The patients/participants provided their written informed consent to participate in this study.

## Author contributions

QW, QY, and RZ contributed equally to this study. All of the authors contributed to the research and development process that resulted in this article. QW, QY, and RZ wrote the manuscript. All of the authors read the manuscript and approved the final manuscript.

## Funding

This work was supported by National Natural Science Foundation of China (81970715); Key Research and Development Program of Sichuan Province (22ZDYF2138).

## Conflict of interest

The authors declare that the research was conducted in the absence of any commercial or financial relationships that could be construed as a potential conflict of interest.

## Publisher’s note

All claims expressed in this article are solely those of the authors and do not necessarily represent those of their affiliated organizations, or those of the publisher, the editors and the reviewers. Any product that may be evaluated in this article, or claim that may be made by its manufacturer, is not guaranteed or endorsed by the publisher.

## Supplementary material

Supplementary Figure 1 Receiver operating characteristic (ROC) curve of cachexia index (CXI) and major complications for determining the cut-off values of low and high CXI groups **(A)** male patients; **(B)** female patients.
