# Supplementary figures and images for: Prognostic value of cachexia index in patients with colorectal cancer: A retrospective study
Source: Front Oncol. 2022 Sep 23;12:984459. doi: 10.3389/fonc.2022.984459 (PMC9540220; doi:10.3389/fonc.2022.984459)

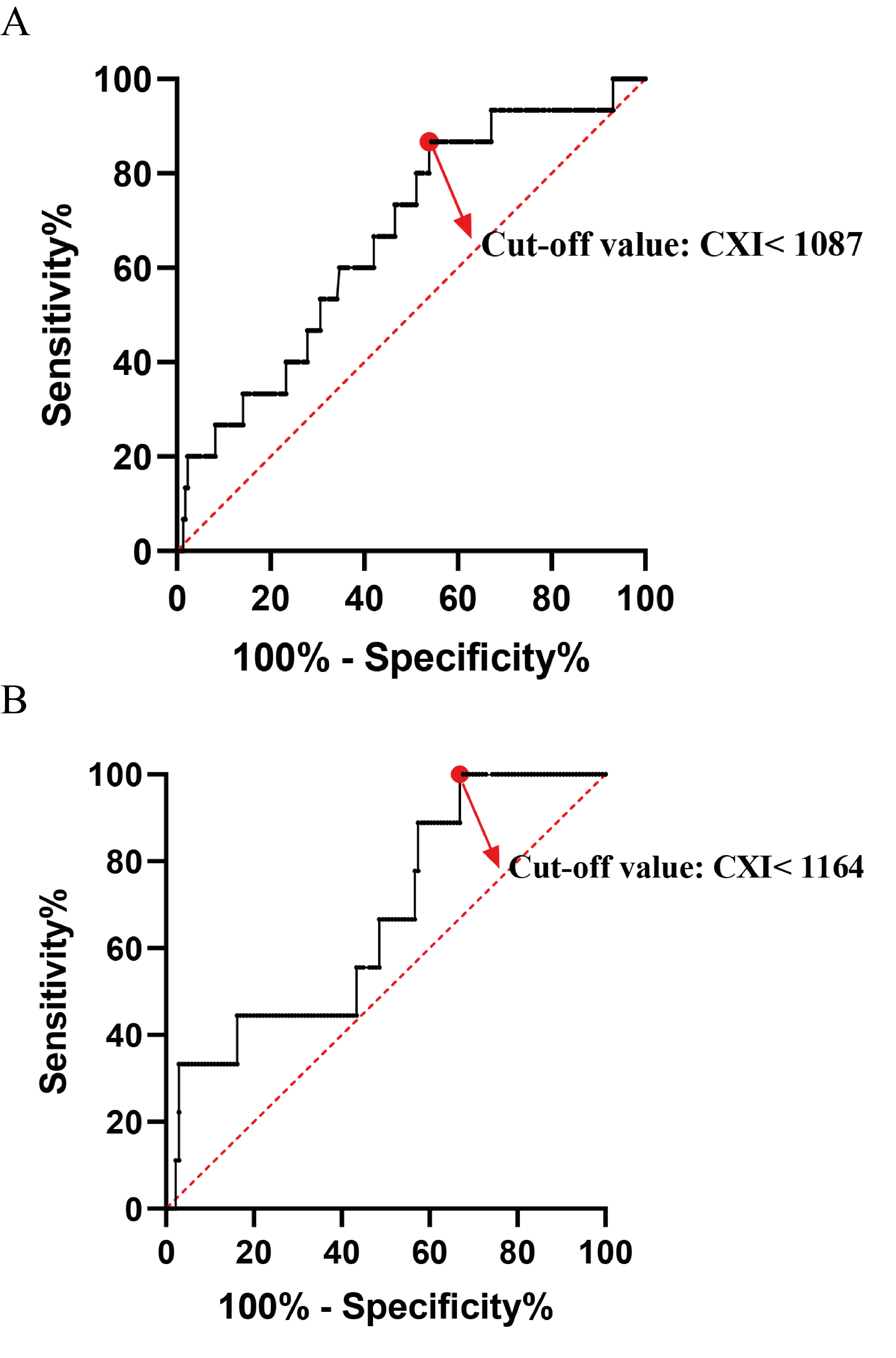

Supplement: Supplementary Figure 1 — Receiver operating characteristic (ROC) curve of cachexia index (CXI) and major complications for determining the cut-off values of low and high CXI groups (A) male patients; (B) female patients. [file Image_1.tiff]
